# Fabrication of Customized Diffractive Optics in under 10 Minutes via Single-Shot Grayscale Projection on a Consumer-Grade DLP System

**DOI:** 10.1021/acsphotonics.5c02622

**Published:** 2026-01-21

**Authors:** Leonid Leites, Reut Orange Kedem, Ori Refael Cohen, Yoav Shechtman

**Affiliations:** † Russel Berrie Nanotechnology Institute, Technion—Israel Institute of Technology, Haifa 3200003, Israel; ‡ Faculty of Biomedical Engineering, Technion—Israel Institute of Technology, Haifa 3200003, Israel; § Faculty of Electrical and Computer Engineering, Technion—Israel Institute of Technology, Haifa 3200003, Israel

**Keywords:** diffractive optical elements (DOEs), grayscale lithography, point spread function (PSF) engineering, 3D localization microscopy, index matching, rapid prototyping

## Abstract

Fabrication of diffractive optical elements (DOEs) is typically slow, costly, and requires specialized expertise, motivating the need for a rapid and accessible alternative. Here, a maskless, cost-effective grayscale lithography approach is introduced for the rapid fabrication of DOEs. The method relies on the single-step projection of a grayscale pattern onto a droplet of UV-curable resin, followed by immersion-oil sealing under near-index-matching conditions. This process reduces the fabrication cycle from several days to about 10 min, remains user-friendly and reliable, and does not require specialized skills. A calibration procedure enables conversion of a phase map into a grayscale pattern without requiring precise direct measurements of the refractive index. The approach demonstrates the fabrication of a vortex plate, Zernike polynomial masks, and phase masks for 3D localization microscopy, all showing strong agreement with simulations. The technique does not require specialized facilities and can be implemented with desktop resin 3D printers, making custom DOE prototyping accessible to a wide range of researchers.

## Graphical abstract

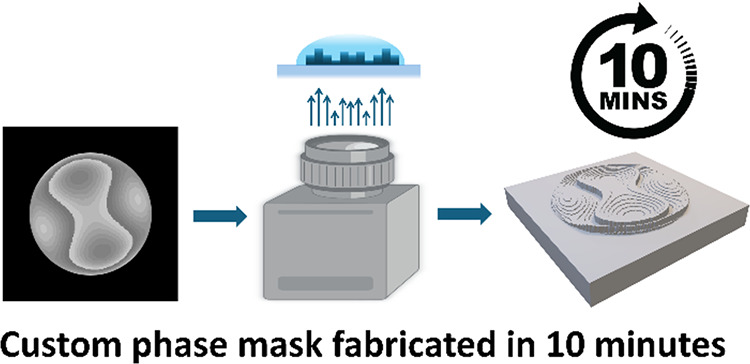

## Introduction

Diffractive optical elements (DOEs) are micro- and nanostructured components that manipulate the phase of light. They enable functionalities such as beam shaping, splitting, and focusing,^1^ and find applications in such areas as microscopy,^2,3^ sensing,^4^ augmented reality^5^ and laser technologies.^6^ Standard DOE fabrication methods such as photolithography, Deep Reactive Ion Etching, and microinjection molding are well suited for mass manufacturing; however, fabrication of custom products using these techniques is generally expensive and time-consuming.^7^

3D-printing offers a potentially appealingly simple and flexible alternative for DOE fabrication; however, most current additive processes rely on layer-by-layer construction, resulting in insufficient resolution for optical applications. Recent work^8,9^ described a DOE fabrication process that combines two media with closely matched refractive indices, allowing for greater height accuracy and error tolerance in optical elements, while enabling the use of standard stereolithography 3D-printing techniques. The method involves 3D-printing a template, converting it into a transparent layer, extracting the first polymer and polymerizing a second layer to form the final DOE. The main fabrication cycle can take in practice several days and is prone to errors due to many manual steps (see Section S4 at the Supporting Information). Thus, it would be desirable to manufacture DOEs directly, without using templates.

DOEs can be manufactured using such techniques as two-photon polymerization (2PP).^10^ For example, recent work^11^ demonstrated direct 2PP 3D-printing with near-index matching, which improved precision in the *Z*-direction and increased fabrication speed thanks to the ability to use larger voxels. Overall, recent advancements in this field enable DOE fabrication within a few hours rather than days. Nevertheless, 2PP requires bulky and expensive equipment, typically operated in clean-room facilities. Among 3D-printing approaches, stereolithography remains both the fastest and most cost-effective method for fabricating custom optical elements. Yet, most studies have focused on refractive optics, aiming to produce smooth lenses^12–19^ rather than DOEs defined by heightmaps with sharp edges and nanoscale variations. A conceptually different approach based on thermocapillary shaping of thin liquid films was demonstrated by Eshel et al.,^33^ enabling rapid fabrication of diffractive optical elements via projected light patterns and subsequent curing. While this method achieves sub-nanometric surface quality and minute-scale fabrication times, it relies on specialized equipment and is fundamentally limited by heat diffusion and convection in the liquid film, which constrain lateral resolution and accurate reproduction of high-spatial-frequency DOE features.

Phase-only DOEs can be represented as heightmaps, i.e., 2.5 dimensional, rather than three-dimensional objects. Thus, a simplified fabrication technique can be implemented by using projection stereolithography 3D-printing systems in which the structure is fabricated through a single grayscale projection onto a droplet of UV-curable resin. This method was first demonstrated by Yuan et al.,^20^ where oscillating the projection optics improved surface quality and enabled rapid, low-cost fabrication of microlens arrays. However, it cannot be directly applied to DOE fabrication, as it lacks precise nanoscale height control.

Here, we present a simple and low-cost method that enables direct fabrication of DOEs within minutes by single-shot grayscale exposure using a maskless lithography approach. The method is implemented on a consumer-grade Digital Light Processing (DLP) 3D printer, which is used solely as a light source. The new method relies on projecting a grayscale pattern onto a droplet of UV-curable resin, followed by applying immersion oil and sandwiching the structure between two glass slides. In our approach, this use of near-index conditions allows the phasemap to be realized with microscale rather than nanoscale relief. We develop a tailored calibration method and demonstrate fabrication of various elements including a vortex plate, Zernike polynomial masks and phase masks for 3D localization microscopy. Overall, our method significantly lowers the barrier to implementing custom DOEs, expanding their use beyond specialized facilities.

## Methods

### Index-Matching Concept

A phase-only DOE can be represented as a heightmap via the following relationship

1 $$\Delta \varphi =\frac{2\pi }{\lambda }\Delta h\Delta n$$

Where Δφ is the phase shift introduced by the DOE, Δ*h* is the local thickness variation of the structure, i.e., the heightmap, and Δ*n* is the refractive index contrast between the DOE material and the surrounding medium. Typically, DOEs fabricated from fused silica using photolithography and placed in air (*n* = 1) exhibit a Δ*n* of about 0.46. In such cases, Δ*h* must be typically on the order of hundreds of nanometers, with surface roughness below 10 nm. Reducing Δ*n* by a factor of 50 enables DOE fabrication with Δ*h* in the range of tens of microns and surface roughness of a few hundred nanometers.^8,9^ Thus, the precision of our method is sufficient to produce a phasemap of high optical quality, using easily accessible DLP projection systems.

### Fabrication Process

The fabrication procedure is illustrated in Figure 1. First, a droplet of UV-curable photopolymer resin is placed on a glass substrate. A single grayscale pattern is then projected onto the resin using a digital light processing (DLP) projector, initiating the solidification process. In a grayscale image, each pixel corresponds to a different light intensity and, therefore, polymerizes the resin to a different depth. Since the droplet thickness (a few millimeters) is much larger than the polymerized height variations, washing away the unexposed resin leaves a heightmap structure attached to the glass. Finally, a droplet of immersion oil is applied, and the structure is sealed by placing another glass slide on top.

**Figure 1:**
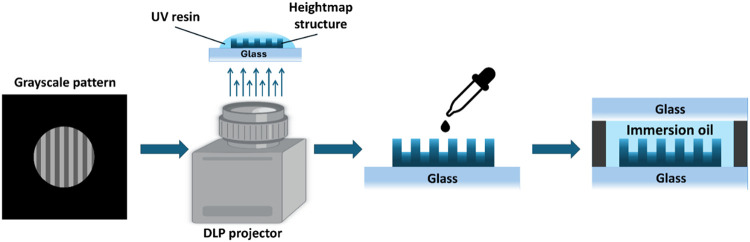
Illustration of the main fabrication steps. First, a heightmap structure is produced by projecting a grayscale pattern onto a droplet of UV resin. Then, a droplet of immersion oil is applied, and the structure is sealed with another slide.

### Materials and Setup

A digital light processing (DLP) 3D printer (MicroSLA, “Micro” model) was employed for all fabrications. The integrated DLP projector is an 8-bit system capable of projecting grayscale values from 0 to 255. A microscopy slide was placed on the printer’s cover glass, and a droplet of photopolymer was deposited on it. The projector has a pixel size of 15 μm, maximum printing area of 15 × 15 mm^2^ and operates at a wavelength of 365 nm. A commercial “MicroSLA clear” resin was used as the photopolymer. The exposure time of the grayscale projection was fixed at 1.5 s, with a light intensity of about 30 mW/cm^2^. Notably, the entire structure is fabricated using a single grayscale exposure, without standard layer-by-layer 3D printing, a build platform, or a resin tank; as a result, the first-layer fabrication typically takes no more than 1–2 min. After fabricating the first layer, we washed it with Isopropanol to get rid of unpolymerized resin in a liquid state and placed a metallic spacer around the heightmap structure to block stray light and to support the glass cover on top. As the second layer we used Immersion Oil Type OVH from Cargile with the viscosity of 46,000 cst. Finally, a droplet of immersion oil was applied, and the structure was sealed with another glass slide. Additional details on the immersion oil and sealing procedures are provided in Sections S2 and S3 of the Supporting Information.

### Calibration Procedure

One of the main challenges is to robustly convert the DOE phase design into a grayscale pattern that can be projected with a defined exposure time and light intensity. Determining the refractive index and the index contrast between the polymer and the immersion oil with high precision is not trivial, as the refractive index strongly depends on the polymerization conditions, including the illumination intensity. As a result, the fabricated heightmap structure may exhibit local variations in refractive index, which are difficult to measure directly.^21^ Here, we suggest a calibration procedure without direct measurements.

We fabricated a set of binary phase diffraction gratings which were designed as two alternating stripes within a circle of 7 mm diameter, each stripe having a width of 300 μm. The odd stripes had a grayscale value 96 for all samples, while the even stripes had values ranging from 96 to 176, for different samples. After fabricating these binary diffraction gratings, the phase difference between the two regions could be measured, allowing us to determine the phase shift corresponding to the difference in grayscale values. The procedure is very similar to the calibration of a liquid-crystal spatial light modulator^22^

After fabrication, the sample was installed into the experimental setup shown in Figure 2a. The phase difference was then calculated from the ratio of the first and zero diffraction orders, which for a binary phase grating with 50% duty cycle gives

2 $$\varphi =\tan^{-1}\frac{\pi }{2}\sqrt{\frac{I_{1}}{I_{0}}}$$

Where *I*_1_, *I*_0_ denote the light intensities of the first and zero diffraction orders, respectively. Figure 2b shows a cross-section of these orders. When the phase is close to zero the zero order dominates, while as the phase approaches π the first order becomes stronger. The derivation of this formula is provided in Section S5 of the Supporting Information.

**Figure 2:**
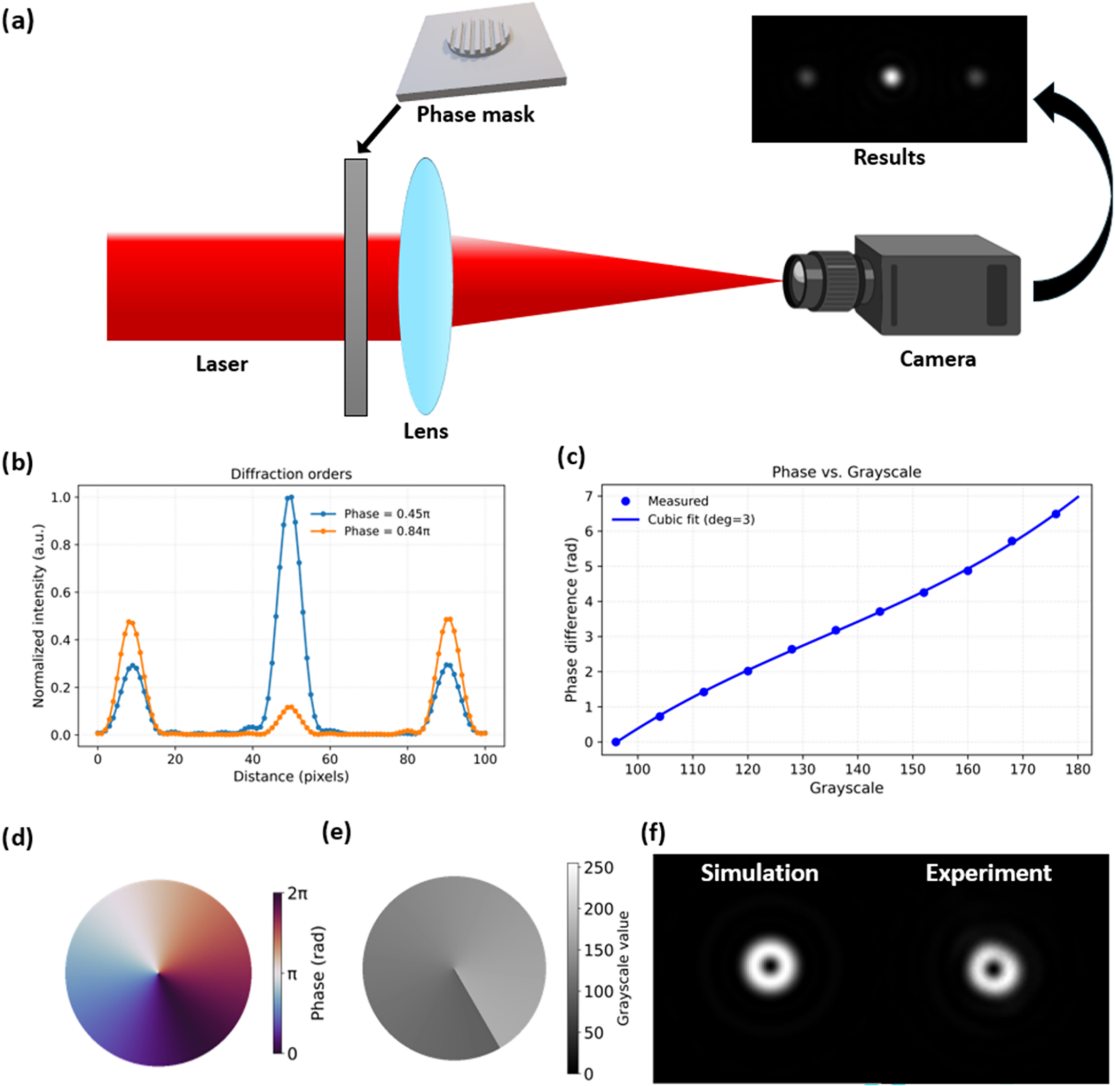
(a) Experimental setup for analyzing DOE performance in the focal plane. Diffraction gratings and vortex phase plates were tested. (b) Measured cross sections of diffraction patterns from gratings with 300 μm stripes, alternating grayscale values of 96/112 and 96/128. (c) Dependence of the phase difference of binary gratings on the grayscale of even stripes (odd stripes fixed at 96). (d) Designed phasemap of the vortex plate. (e) Grayscale map sent to the DLP projector. (f) Comparison of simulated and experimental results of the vortex plate.

By summing the pixel values around the first and zero order spots, we obtained *I*_1_ and *I*_0_ for each sample and derived the dependence of the phase difference on the grayscale value of the projected pattern, as shown in Figure 2c. We achieved a maximum phase difference of 2π, which is sufficient for fabricating a wide range of phase DOEs. The maximum attainable phase difference depends on the maximum height difference, and, therefore, on the energy dose of a grayscale pattern. With the increase of the energy dose, effects such as scattering, self-focusing and overcuring become critical. It is recommended to keep the height difference in the range of few hundred microns, which can give a maximum phase difference of about 8π–10π. Photopolymer resin should be stored in a dark place at room temperature, and the projection energy dose should be kept the same. Under these conditions, the same calibration procedure can be used for at least 6 months and any DOE design with a given phase range based on this calibration can be fabricated. If a different material, light source, or exposure parameters are used, the calibration should be repeated.

## Results and Discussion

### Vortex Phase Plate Fabrication

After building the calibration curves, we manufactured a vortex phase plate, which forms a donut-shaped intensity profile at the focal plane (Figure 2d,e). To test the sample, we used the same experimental setup as the one used for the diffraction grating analysis (Figure 2a). Analysis of the focal spot revealed that the fabricated phase mask produced higher phase values than designed, which we attribute to edge smoothing of the binary gratings during calibration. Comparing with simulations, we found that scaling the theoretical phasemap by a factor of 0.9 yields good agreement (Figure 2f). Additional calibration tests with narrower gratings confirmed stronger smoothing effects, supporting this explanation (See Supporting Information Figure S1).

### Height and Phase Profile Characterization

To evaluate a typically achievable geometry of the height map structure before the index-matching process, we manufactured an element consisting of alternating concentric rings of different thicknesses, where odd rings provide a phase shift of 0 and even rings provide a phase shift of π. Figure 3a shows the fabricated height map observed with brightfield microscopy. Confocal microscopy (Sensofar S Neox 3D Optical Profiler) was then used to measure the height map (Figure 3b). It can be observed that the edges of the rings are not perfectly sharp; smoothing effects are present, as suggested in the previous section. A phase difference of π corresponds to a height difference of about 23 μm, which, according to formula [Disp-formula eq1], gives a refractive index contrast of approximately 0.012. However, this estimation does not account for a possible dependence of the refractive index on the grayscale values and, therefore, it is more reliable to use the calibration procedure described earlier. Figure 3c shows the surface roughness profile, from which we estimate that root-mean-square error (Rz) is approximately 0.21 μm. This corresponds to an effective optical surface roughness of about 4 nm after near-index matching.

**Figure 3:**
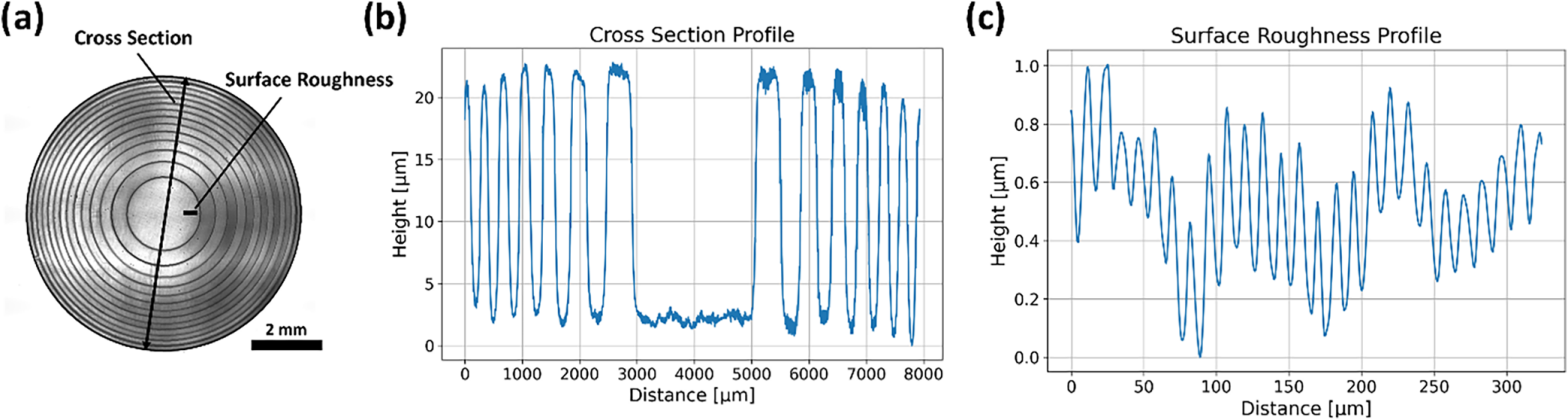
(a) Brightfield microscopy image of the test sample. (b) Cross-section of height profile measured by confocal microscopy. (c) Surface roughness profile over a small section.

### Zernike Polynomial Phase Mask Fabrication

Zernike polynomials provide a convenient test case since they form a standard basis for describing wavefront aberrations and are widely used in optical design. Zernike masks are used in ophthalmology,^23,24^ where combinations of these modes are applied to represent and correct ocular aberrations or to calibrate ophthalmic devices. Although modern studies often rely on spatial light modulators (SLMs),^25,26^ compact static phase masks could be attractive as a low-cost alternative for education, demonstrations, and potentially for clinical use where bulky SLM setups are impractical.

For the demonstration purposes we fabricated three phase masks of fifth order Zernike polynomials (Z_5_^1^, Z_5_^3^, Z_5_^5^) with a 3 mm diameter, which corresponds to the secondary coma, trefoil and pentafoil aberrations. Imaging was conducted using the setup shown in Figure 2a. The results are presented in Figure 4a, showing a strong agreement between simulation and experiment.

**Figure 4:**
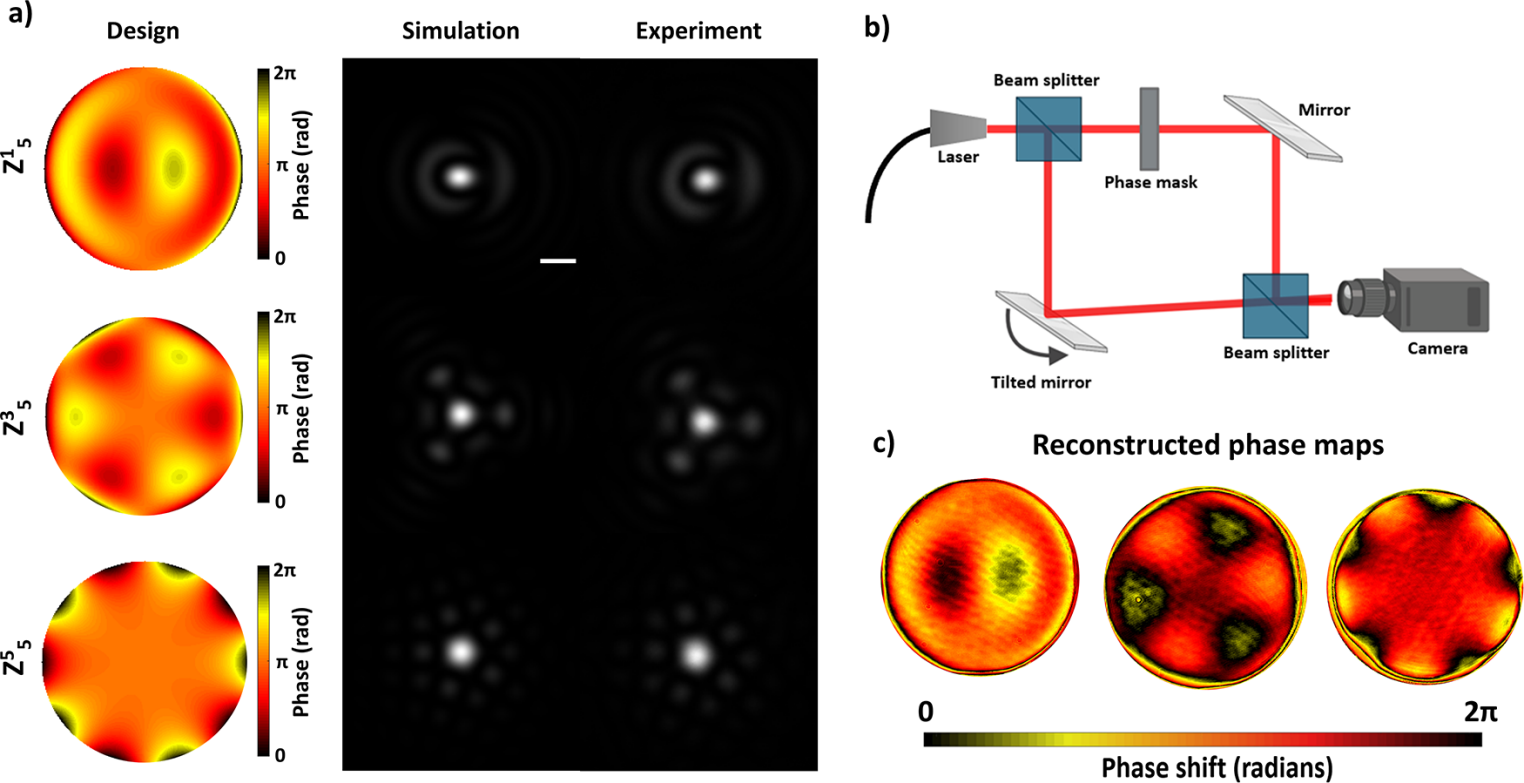
(a) Designed phase maps, simulated and experimental imaging results of three-fifth-order Zernike polynomials (Z_5_^1^, Z_5_^3^,Z_5_^5^; Noll indexing) placed in the Fourier plane. Scale bar: 80 μm (b) Off-axis Mach–Zehnder holography setup used for phase measurements (c) Reconstructed phase maps. Contrast was adjusted for visibility.

Additional characterization was performed using an off-axis holography setup based on a Mach–Zehnder interferometer to measure the phase profile (Figure 4b). The laser beam is split into a reference arm and an object arm containing the phase mask, and the resulting interference pattern is recorded after recombination. A slight tilt in one of the mirrors introduces an off-axis angle, enabling separation of the interference terms in Fourier space and unambiguous phase retrieval. The reconstructed phase exhibits the expected modulation pattern, with minor deviations attributed to residual optical aberrations (Figure 4c).

### Phase Mask Fabrication for Point Spread Function Engineering

Our technology finds practical applications in the field of 3D localization microscopy. By introducing specific phase masks in the back focal plane of a microscope, it is possible to modify the shape of the point spread function (PSF) and thus encode z-information more efficiently.^3,27,28^ Analyzing images of point sources (e.g., fluorescent beads or nanoparticles) then allows their 3D position to be determined within a range of a few microns to tens of microns, which has been used in various applications in super-resolution microscopy and single particle tracking.^29^ In this work we used a 4F extension to a Nikon Ti2-E optical microscope and placed a phase mask in the Fourier plane (see Figure 5a). However, if necessary, fabricated phase masks can also be inserted under the objective using an adaptor, without the need to disassemble the microscope.^30^

**Figure 5:**
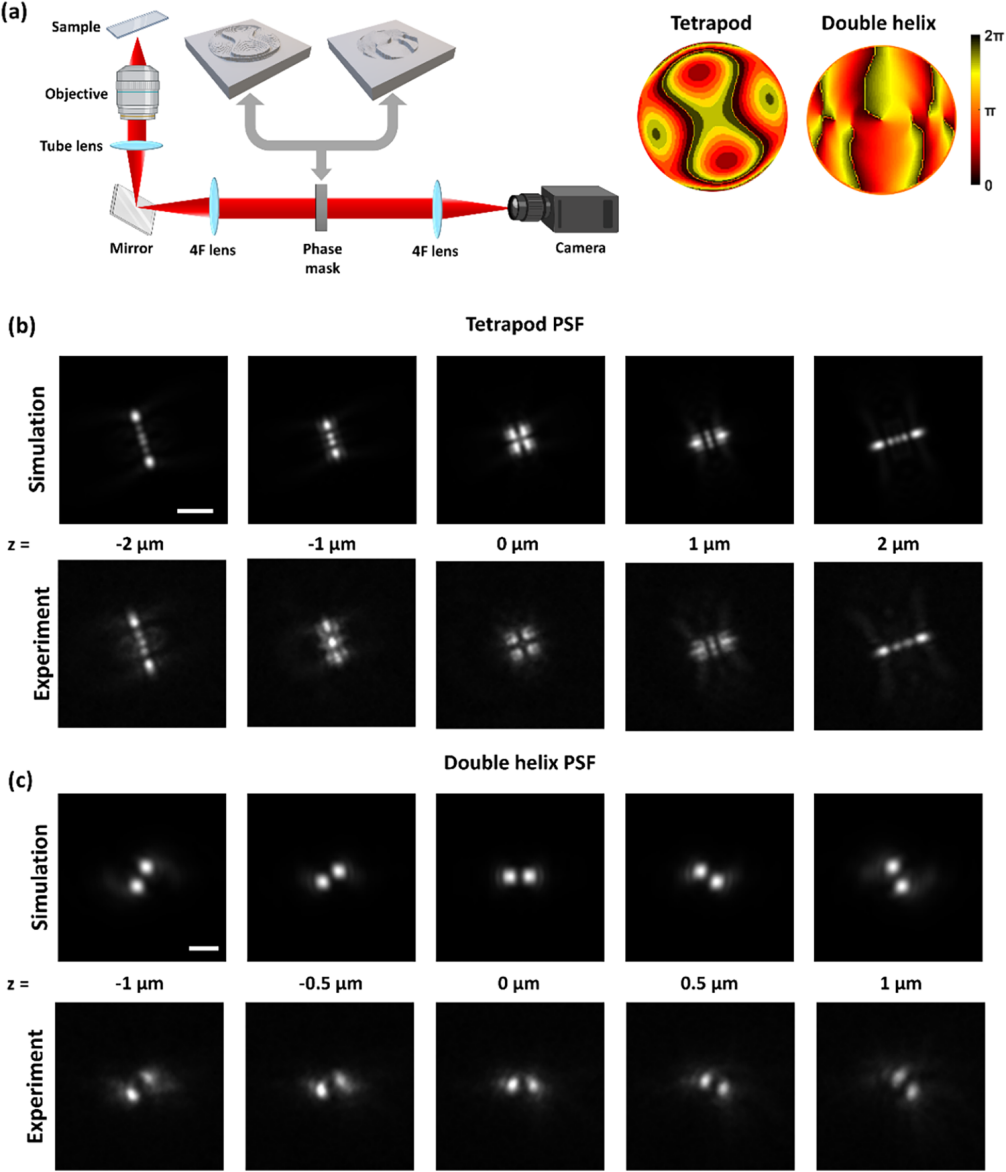
Fabricated phase masks for 3D PSF engineering. (a) 4f microscopy setup with a phase mask in the Fourier plane, along with phase maps of the Tetrapod (left) and Double Helix (right) masks. (b) Simulated and experimental PSFs of the Tetrapod mask across a 4-μm axial range (scale bar: 3 μm). (c) Simulated and experimental PSFs of the Double Helix mask across a 2-μm axial range (scale bar: 3 μm).

The first phase mask we fabricated was a Tetrapod phase mask, which generates a PSF with a characteristic four-lobed shape.^29^ We then measured a *z*-stack of a fluorescent bead (0.1 μm in size with 680 nm wavelength of emission peak) over a 4-μm axial range (see Figure 5b). Second, we fabricated a Double Helix phase mask, which generates a PSF with two lobes that rotate as the emitter moves axially,^27^ and measured a *z*-stack over a 2-μm range (see Figure 5c). Both phase masks had a diameter of 5 mm and were characterized using a 100×, 1.49 NA oil-immersion objective. All results show good agreement with simulations, with deviations attributable to smoothing effects during fabrication and to optical aberrations of the experimental setup. These phase masks are readily usable for PSF engineering and 3D localization microscopy.

## Conclusion

Here we demonstrated a DOE fabrication method at unprecedented speed, that does not require high costs or advanced skills. The whole process from a phase-map file to a fully functional element takes ∼10 min. In principle, it can be implemented with any desktop DLP/LCD 3D printer if grayscale projection is supported. Alternatively, a simple custom setup with a DLP or LCD light source can be built. This opens the way for researchers to use this method to improve R&D through rapid prototyping without relying on external services. Currently, the main limitation of this method is the deviation of the fabricated height map from the initial design, which arises from the finite optical resolution of the light source as well as chemical effects such as free-radical diffusion within the photopolymer. These effects can be minimized by optimizing the material properties and exposure dose, using a light pattern with a smaller pixel size, and estimating the fabrication kernel to predict, account for, and potentially compensate for geometrical deviations at the design stage.^31^ However, the fabrication of DOEs containing numerous abrupt phase jumps or very sharp lateral features may still represent a fundamental limitation of the present approach and will be addressed in future work. Nevertheless, the method is well suited for DOEs with continuous or slowly varying phase profiles, where such smoothing effects have a minimal impact on performance. A second limitation is that the method is not suitable for high-power applications; this can be addressed by using composite photopolymers that can be sintered into fused silica after printing in an oven.^32^ Taken together, the method significantly lowers the entry barrier for rapid prototyping of diffractive optical elements in standard laboratory settings.

## Supplementary Material



## Notes

Analysis of diffraction grating behavior for different widths, long-term stability characterization, a description of the sealing procedures, and a mathematical derivation of the phase-intensity relationship for a binary phase grating (PDF)
