# Supplementary material for: Fabrication of Customized Diffractive Optics in under 10 Minutes via Single-Shot Grayscale Projection on a Consumer-Grade DLP System
Source: ACS Photonics. 2026 Jan 21;13(3):808–14. doi: 10.1021/acsphotonics.5c02622 (PMC12880098; doi:10.1021/acsphotonics.5c02622)
Supplement: Supplementary file 1 [file ph5c02622_si_001.pdf]

# Supplementary information for:

# Fabrication of customized diffractive optics in under 10 minutes via single-shot grayscale projection on a consumer-grade DLP system

**Leonid Leites[1], Reut Orange Kedem[1], Ori Refael Cohen[1] and Yoav Shechtman[2,3]***

1. *Russel Berrie Nanotechnology Institute, Technion – Israel Institute of Technology, Haifa, Israel, 3200003*
2. *Faculty of Biomedical Engineering, Technion—Israel Institute of Technology, Haifa, Israel, 3200003.*
3. *Faculty of Electrical and Computer Engineering, Technion—Israel Institute of Technology, Haifa, Israel, 3200003.*

*yoavsh@technion.ac.il

This PDF contains:

Pages: 12

Figures: 5

Formulas: 9

**Contents:**

S1. Influence of Stripe width on Phase Difference

S2. Long-term stability of the DOE

S3. Sealing procedure

S4. Workflow previously used in near-index-matching additive manufacturing-based DOE fabrication

S5. Derivation of the phase-intensity relationship for a binary phase grating (Eq. 2)

## S1. Influence of Stripe width on Phase Difference

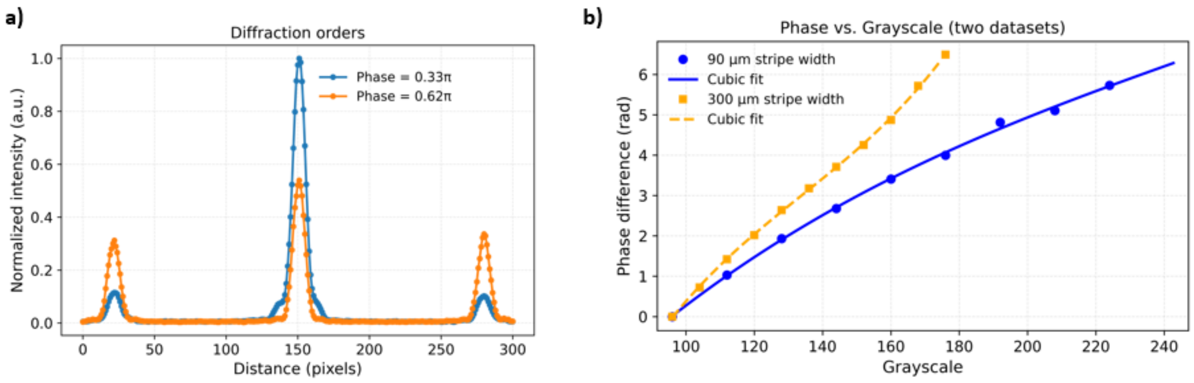


**Fig. S1** a) Cross-sections of the diffraction patterns measured from gratings with 90 µm stripe width and grayscale projection values of 96 and 112. b) Calibration curves obtained from diffraction gratings with stripe widths of 90 µm and 300 µm, respectively.

Our calibration process, i.e. the translation from grayscale illumination values to phase pattern, is based on measuring diffraction from a grating. However, the properties of this diffraction pattern vary as a function of the grating period, due to finite fabrication resolution. Specifically, at high frequencies, the grating contrast is decreased. To further characterize this effect, we fabricated a set of calibration gratings with narrower stripes. Figure 1Sa shows cross-sections of the diffraction patterns for gratings with grayscale values of 96/112 and 96/128. It is useful to compare this with Fig. 2b in the main manuscript: all other parameters are identical, but the reduced stripe width results in smaller changes in the intensities of the zero and first diffraction orders. Figure 1Sb presents two calibration curves obtained from gratings with stripe widths of 90 µm and 300 µm, respectively. The data show that the phase difference for gratings with 300 µm stripes increases more rapidly, as relative smoothing effects are smaller and diffraction is more efficient. The curve with 300 µm was chosen to fabricate all DOEs in the paper.

## S2. Long-term stability of the DOE

Using immersion oil as a second layer allows simple and fast deposition and gives high-quality results, but may raise questions about the potential long-term stability and durability of the DOE. This process requires detailed long-term characterization, but preliminary tests show that no significant changes occur over 40 days. Figure S2 shows measured Z-stacks of the fabricated tetrapod phase mask immediately after fabrication and 40 days later, indicating no or only minor differences. Data sheet from Cargille for Immersion Oil Type OVH indicates that the refractive index change of the immersion oil due to evaporation is very low (expected $\Delta n \approx 0.000001$), so this factor can be neglected. The only potential issue is penetration of the oil into the first layer over time, but this effect is expected to be insignificant for two reasons. First, the first layer is very thin and undergoes an additional one minute UV post-curing step, resulting in a high degree of polymerization and low porosity. Second, the viscosity of the oil is very high (46,000 cSt) and, therefore, flow in any direction is very slow.

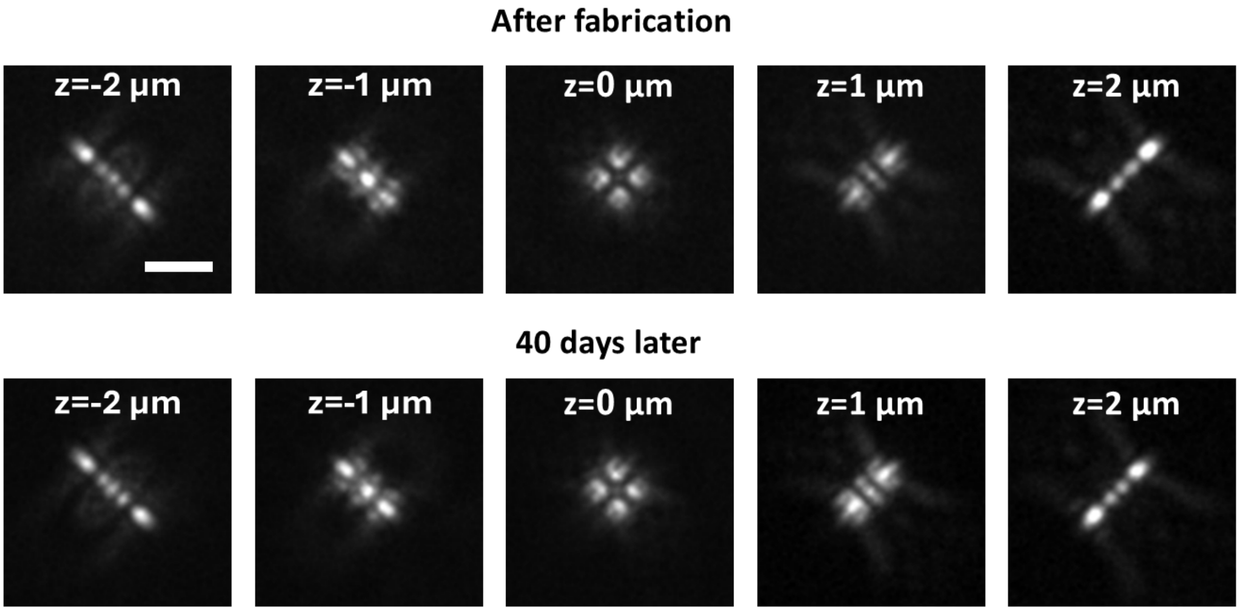


**Fig. S2**. Experimental PSFs of the Tetrapod mask across a 4-µm axial range after the fabrication and 40 days later. Phase mask was manufactured using 3D-Printing for the first layer and immersion oil for the second layer. Scale bar: 3 µm

At the same time, fabrication of fully solid DOEs is possible by using appropriate materials for the second layer. For example, UV-curing resins are not recommended for the second layer, as non-uniform shrinkage leads to refractive index gradients and internal stress, which degrades PSF quality. Two potentially suitable alternatives are optical gel (Optical Gel nD 1.52, Cargille), which is a solid-state material that does not require a solidification process and therefore exhibits zero shrinkage, and a thermoplastic material (Meltmount™ 1.539, Cargille). The solidification process of the thermoplastic is reversible and occurs during cooling from approximately 100 °C to room temperature. If the cooling process is sufficiently slow, shrinkage is minimal, although this may slightly increase the fabrication time. Further characterization of these materials is a part of future work.

## S3. Sealing procedure

Sealing procedures can be implemented in different ways depending on the specific application; here we describe the two main approaches used in this work. In the first method, standard microscopy slides are used as substrates. A key point is that the lateral size of a microscopy slide (76 mm) is much larger than the diameter of the phase mask (typically 5-15 mm). After fabrication and removal of residual unpolymerized resin, a metal washer with a thickness slightly larger than that of the phase mask is placed around it. The washer serves both as a light-blocking aperture and as a mechanical spacer supporting the second glass substrate.

UV-curable adhesive (Norland Optical Adhesive 68 or similar) is then applied along the edges of the microscopy slide. A small amount of immersion oil is deposited onto the phase mask; the exact volume is not critical, as long as the entire mask is covered. The second glass is then placed on top and gently pressed, causing the immersion oil to spread around the metal washer. UV light is subsequently used to cure the adhesive at the edges. Because the glass substrate is much larger than the phase mask, the immersion oil typically does not come into contact with the UV adhesive. Bubble formation is usually not a significant issue, as no mixing process is involved. Any occasional bubbles can be displaced with a pipette or removed by forming a thin oil layer and pressing the glass.

An important aspect of this sealing approach is the very high viscosity of the immersion oil (46,000 cSt). Even if nanoscale or submicron gaps (<1 µm) are present between the metal washer and the glass, leakage through these gaps is kinetically suppressed. The combination of a long and narrow leakage path, capillary forces, and the extremely high viscosity of the oil results in negligibly slow flow, making leakage insignificant on experimental time scales. Typical process as well as the final structure is shown in figure S3.

We recommend using this sealing method for long calibration processes or for tests when fabrication parameters and results are uncertain. Although it provides high-quality results and can be used in final experiments, handling may be slightly inconvenient due to the size of the glass substrate.

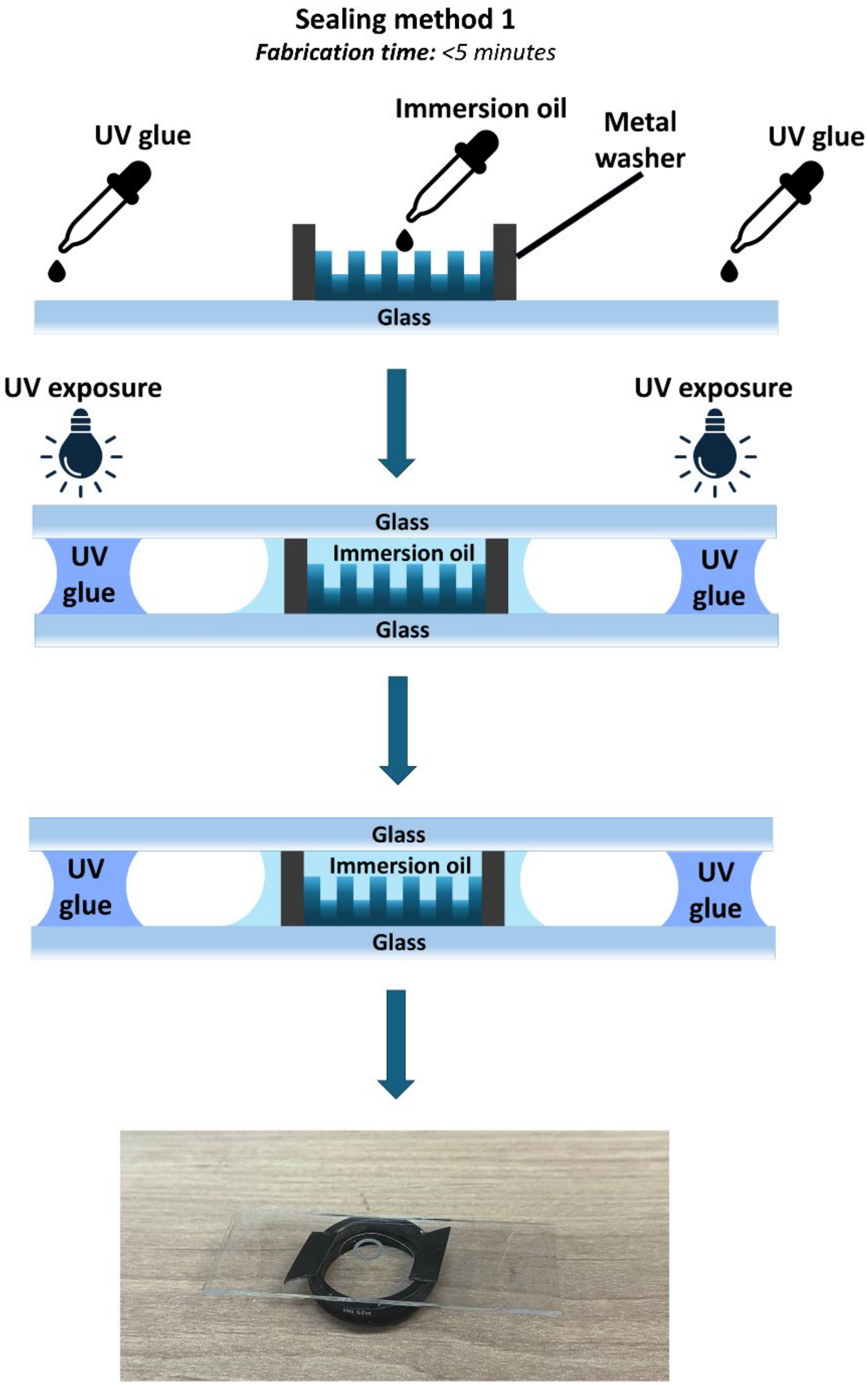


**Fig. S3.** Sealing method 1. Schematic of the sealing procedure for a viscous immersion oil second layer using a metal washer and UV-curable adhesive

The second sealing procedure allows the fabrication of more compact structures that are easier to use in final experiments, but it may require slightly more expensive custom-sized glass substrates and 3D-printed parts. In this method, we used a Bambu Lab X1 FDM 3D printer to fabricate a custom sealing structure. Since these parts can be prepared in advance in large quantities, they are typically not included in the fabrication time. In this work, we used 25-mm-diameter round glass substrates, although smaller sizes can be used for more compact designs if needed.

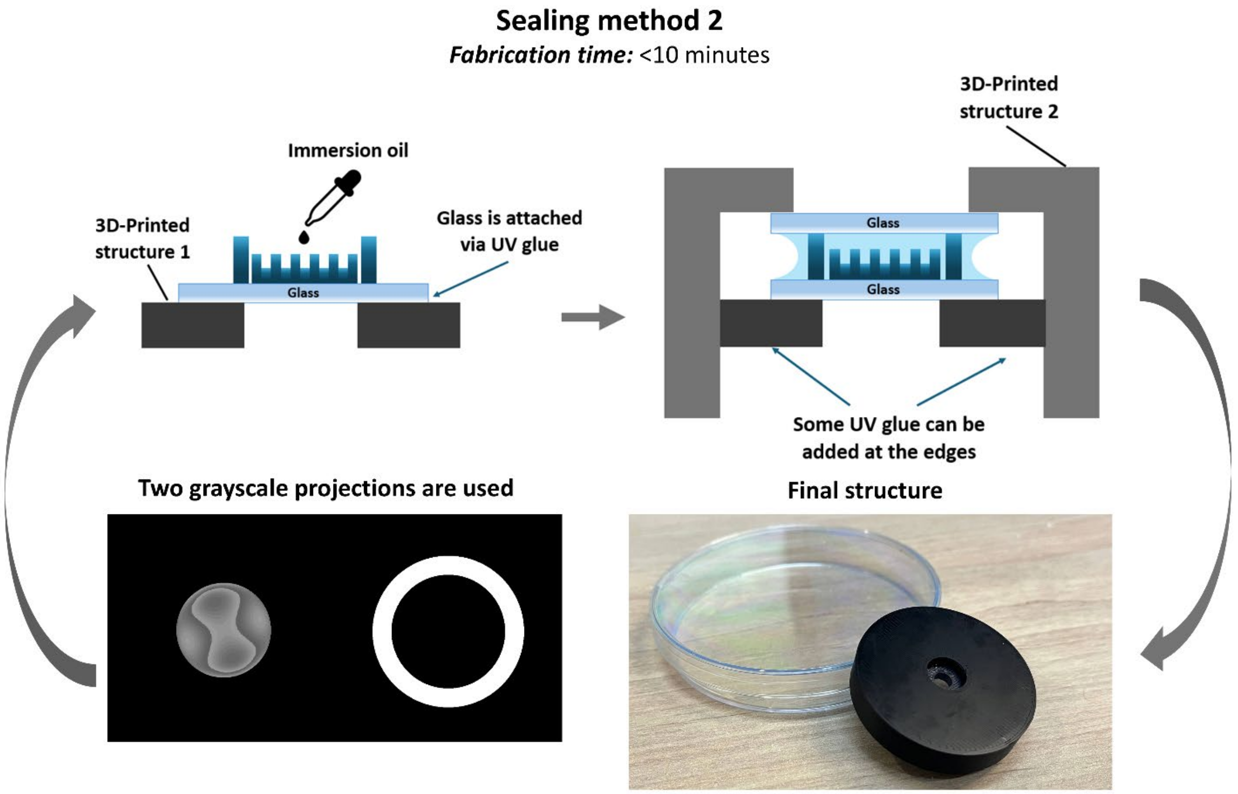


**Fig. S4**. Sealing method 2. Schematic of a compact sealing procedure for a viscous immersion oil second layer using mechanically confined 3D-printed holders

First, the phase mask is fabricated by grayscale projection. Two projections are used: the first defines the height-map structure of the phase mask, and the second forms surrounding walls to improve mechanical sealing. The fabricated structure is then attached to a 3D-printed ring with an inner diameter matching that of the phase mask. A small amount of immersion oil is deposited, after which a glass substrate is placed on top. The assembly is subsequently inserted into a second 3D-printed holder with a ring-like geometry that tightly accommodates the first structure. If the mechanical fit is not perfect, a small amount of UV-curable adhesive can be applied at the edges.

In this approach, sealing is achieved primarily through mechanical confinement, and the UV adhesive does not directly contact the immersion oil or the optical region. The amount of

immersion oil should be minimized and concentrated around the phase mask area; however, this requirement is not strict. Only in extreme cases, excessive oil combined with strong pressure may cause leakage onto the upper glass surface.

The entire process is illustrated in Fig. S4 and can be used for final experiments, as the resulting structures are more compact and convenient, for example, for use with standard mounting rings in optical setups.

## S4. Workflow previously used in near-index-matching additive manufacturing-based DOE fabrication

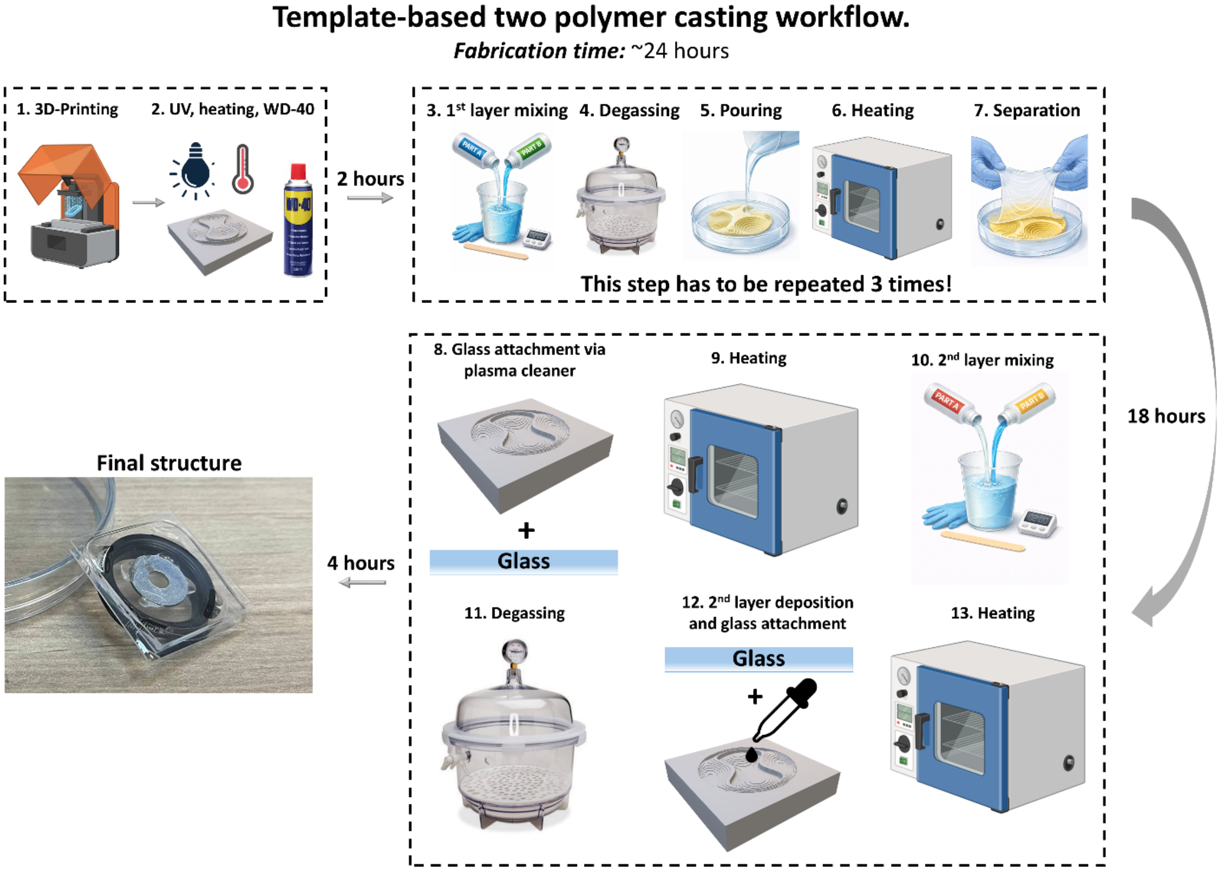


**Fig. S5.** Fabrication workflow of the method developed previously in our research group[1]. All fabrication steps with time are illustrated to show the complexity of the process in comparison with the method proposed in the current research work

To better demonstrate the improvements in contrast with the method previously developed in our research group[1], we show the complete fabrication workflow of this method in Fig. S5. The method consists of three major stages.

First, the template is 3D-printed using a standard stereolithography (SLA) layer-by-layer process, which takes approximately 1 hour. The printed structure then undergoes UV post-curing for 30 minutes and additional thermal curing in an oven at ~70 °C for 30 minutes to increase the degree of polymerization and remove residual unpolymerized resin prior to molding. This stage takes about 2 hours in total

The second stage consists of molding the polymer on the fabricated template. In this protocol, a thin layer of WD-40 silicone oil is deposited on the 3D-printed template, which increases polymerization inhibition at the interface but significantly reduces adhesion and facilitates separation of the first layer. Although this approach is the most time-consuming, it is also the most robust, minimizing fabrication issues during the first-layer release. Firstly, the two components of the polymer are mixed and placed in a vacuum chamber to remove mixing-related bubbles. The polymer is then molded upon the template in a Petri dish, cured in an oven for at least 4 hours, and separated. Usually, this stage has to be repeated three times for each new template, because after the first iteration, polymer inhibition is typically observed at the interface, and polymer residues remain attached to the template. After the second iteration, these residues are removed during separation. Finally, after the third iteration, the polymer is easily removed and properly polymerized. At this point, the first DOE layer is fabricated. The total fabrication time of this stage is at least 18 hours.

The third stage consists of attaching the glass to the first layer via plasma cleaning, heating it in the oven, preparing the second polymer, degassing in a vacuum chamber, depositing onto the second layer, attaching the glass, and finally heating in the oven for about 2 hours (a shorter heating time is typically sufficient due to the small layer thickness).

As a result, the typical fabrication time is about 24 hours and involves substantial manual work, meaning that mistakes can require the entire process to be repeated. In addition, this method is more challenging for fabricating thin structures, as mechanical separation of a thin polymer layer from the template can be difficult

## S5. Derivation of the phase-intensity relationship for a binary phase grating (Eq. 2)

Within one period of the grating, the complex transmission function can be written as:

$$t(x) = \begin{cases} 1, & 0 \leq x < \Lambda/2 \\ e^{i\varphi}, & \Lambda/2 \leq x < \Lambda \end{cases} \quad \text{(S1)}$$

This function is periodic with period Λ.

When the grating is imaged in the focal plane of a lens, the focal-plane diffraction pattern corresponds to the Fourier transform of the grating's complex transmission function. Consequently, the discrete diffraction orders are proportional to the Fourier coefficients of $t(x)$:

$$A_m = \frac{1}{\Lambda}\int_0^{\Lambda} t(x)\ e^{-i2\pi m x/\Lambda}\,dx \quad \text{(S2)}$$

For $\mathrm{m} = 0$ the integral gives

$$A_0 = \frac{1}{\Lambda}\left(\int_0^{\Lambda/2} 1\ dx + \int_{\Lambda/2}^{\Lambda} e^{i\varphi}\ dx\right) = \frac{1}{2}\left(1+e^{i\varphi}\right) \quad \text{(S3)}$$

The corresponding intensity is

$$I_0 = |A_0|^2 = \frac{1}{4}|1 + e^{i\varphi}|^2 = \frac{1}{2}(1 + cos\varphi) \quad \text{(S4)}$$

For the first diffraction order ($\mathrm{m} = \pm 1$), the Fourier coefficient becomes

$$A_{\pm 1} = \frac{1}{\Lambda}\left(\int_0^{\Lambda/2} e^{-i2\pi x/\Lambda}\ dx + \int_{\Lambda/2}^{\Lambda} e^{i\varphi}\ e^{-i2\pi x/\Lambda} dx\right) \quad \text{(S5)}$$

These integrals give

$$A_{\pm 1} = \frac{1}{\pi}\left(1-e^{i\varphi}\right) \quad \text{(S6)}$$

The corresponding intensity is

$$I_1 = |A_{\pm 1}|^2 = \frac{1}{\pi^2}|1 - e^{i\varphi}|^2 = \frac{2}{\pi^2}(1 - cos\varphi) \quad \text{(S7)}$$

Taking the ratio of the first-order and zeroth-order intensities gives

$$\frac{I_1}{I_0} = \frac{4}{\pi^2} tan^{\,2}\left(\frac{\varphi}{2}\right) \quad \text{(S8)}$$

Therefore

$$\varphi = 2tan^{-1}\left(\frac{\pi}{2}\sqrt{\frac{I_1}{I_0}}\right) \tag{S9}$$

which corresponds to Eq. (2) in the main text.

# References

[1] R. Orange Kedem, N. Opatovski, D. Xiao, B. Ferdman, O. Alalouf, S. Kumar Pal, Z. Wang, H. von der Emde, M. Weber, S. J. Sahl, A. Ponjavic, A. Arie, S. W. Hell, Y. Shechtman, *Light Sci. Appl.* **2023**, *12*, 222.
